# *Plasmodium falciparum *enolase: stage-specific expression and sub-cellular localization

**DOI:** 10.1186/1475-2875-8-179

**Published:** 2009-07-30

**Authors:** Ipsita Pal Bhowmick, Nirbhay Kumar, Shobhona Sharma, Isabelle Coppens, Gotam K Jarori

**Affiliations:** 1 Department of Biological Sciences, Tata Institute of Fundamental Research, Homi Bhabha Road, Colaba, Mumbai-400005, India; 2 Department of Molecular Microbiology and Immunology, Johns Hopkins Bloomberg School of Public Health, Baltimore, Maryland, USA

## Abstract

**Background:**

In an earlier study, it was observed that the vaccination with *Plasmodium falciparum *enolase can confer partial protection against malaria in mice. Evidence has also build up to indicate that enolases may perform several non-glycolytic functions in pathogens. Investigating the stage-specific expression and sub-cellular localization of a protein may provide insights into its moonlighting functions.

**Methods:**

Sub-cellular localization of *P. falciparum *enolase was examined using immunofluorescence assay, immuno-gold electron microscopy and western blotting.

**Results:**

Enolase protein was detected at every stage in parasite life cycle examined. In asexual stages, enolase was predominantly (≥85–90%) present in soluble fraction, while in sexual stages it was mostly associated with particulate fraction. Apart from cytosol, enolase was found to be associated with nucleus, food vacuole, cytoskeleton and plasma membrane.

**Conclusion:**

Diverse localization of enolase suggests that apart from catalyzing the conversion of 2-phosphoglycericacid into phosphoenolpyruvate in glycolysis, enolase may be involved in a host of other biological functions. For instance, enolase localized on the merozoite surface may be involved in red blood cell invasion; vacuolar enolase may be involved in food vacuole formation and/or development; nuclear enolase may play a role in transcription.

## Background

In recent years, it is being realized that many of the house-keeping metabolic enzymes participate in a host of other biological functions inside the cell. It is increasingly becoming apparent that the ability of a protein to 'Moonlight' i.e. to have multiple and sometimes vastly unrelated functions embedded within one polypeptide chain, is a general strategy to enhance the number of protein functions that are encoded by the genome [1]. Many of the metabolic enzymes, specifically the glycolytic ones from different organisms have diverse functions in addition to their role in glycolysis. For example, hexokinase2 in yeast is involved in transcriptional regulation [2], glyceraldehyde 3-phosphate dehydrogenase functions in tubulin binding, nuclear RNA export, phosphorylation, membrane fusion, and transcriptional regulation [3-5], glucose-6-phosphate isomerase in cell motility and proliferation [6] and aldolase binds actin and support protein trafficking to the plasma membrane [7,8]. Thus, functional moonlighting for many of these house-keeping proteins, seems to be a general phenomenon [9].

Glycolytic enzymes play important roles in *Plasmodium *biology. Intra-erythrocytic stages of *Plasmodium falciparum *lacks functional TCA cycle and solely rely on glycolysis for their energy needs [10-12]. The level of glycolytic flux in parasite infected cells is ~100 fold greater than that of uninfected red blood cells [13,14] and the activity of some of the glycolytic enzymes (enolase, pyruvatekinase and hexokinase) is greatly up-regulated [15]. In recent years, glycolytic enzymes have also been shown to perform non-glycolytic functions in apicomplexan parasites. In *Toxoplasma *and *Plasmodium*, aldolase has been implicated in host cell invasion through its interaction with actin and surface adhesion molecules [7,8,16]. Interestingly, glycolytic and non-glycolytic functions of aldolase are made mutually exclusive as adhesins bind at the active site resulting in loss of catalytic activity. Similarly, glyceraldehydes 3-phosphate dehydrogenase (GAPDH) has also been shown to perform certain non-glycolytic functions in *P. falciparum *[17]. Due to their importance in *Plasmodium *for energy production and other physiological functions, glycolytic enzymes have been termed as important therapeutic targets and validated in the new large scale ventures for anti-malarials [12,18-21].

Enolase (2-Phospho-D-glycerate hydrolase; EC 4.2.1.11) is one of the three glycolytic enzymes, whose levels are highly elevated in parasite infected red blood cells (RBC) (about 15-fold) as compared to the uninfected cells [15]. Recently, this glycolytic enzyme has also been reported to have diverse biological functions in different organisms [22-26]. Thus, enolases, which have been well characterized for their catalytic function in glucose metabolism, are no longer considered to be the house-keeping enzymes only. Enolase is better described as a multifaceted protein with multi-tasking abilities at diverse sub-cellular locations [24]. Recent studies have shown that in many pathogenic species and in different cell types, enolase is present on the cell wall, cell membranes and in the cell nucleus. The unusual location of enolase has been reported in the apicomplexan parasites viz. *Plasmodium yoelii *[27], *Toxoplasma gondii *[28] and *Eimeria tenella *[29]. In *T. gondii*, there are two different isozymes (Eno1 and Eno2), which have been demonstrated to exhibit stage specific expression. A comparison of mRNA expression of glycolytic genes between tachyzoite vs *'in vitro*' bradyzoite has shown that the two enolase genes are the only glycolytic genes whose expression is regulated in a stage specific manner. Eno1 is strongly up-regulated (~1450 fold) in bradyzoite while all other glycolytic transcripts were elevated only by 4- to 8-fold. At protein level, ENO1 is specifically expressed in bradyzoites, while ENO2 expression is specific to tachyzoite stage. Location of two enolase isozymes in the nucleus of actively developing/dividing parasites has led to the suggestion that these proteins may play a role in controlling some nuclear activities during stage differentiation [28,30]. Among the genes that code for glycolytic enzymes in *T. gondii*, silencing of ENO2 had the most effect on parasite growth [31]. Such observations of stage specific isozyme expression, nuclear localization and growth inhibition on loss of function of this glycolytic enzyme suggest interesting nuclear function for this protein in *T. gondii*. Since *Plasmodium *has only one gene for enolase, it is likely that such non-glycolytic function(s), if mediated, may be embedded in a single enolase protein.

Evidence has emerged from the recent experiments that enolase may have moonlighting functions in *Plasmodium*. Preliminary immunofluorescence studies on *P. yoelii *showed the presence of enolase in parasite nucleus and on merozoite cell surface. Further evidence for novel surface function(s) for enolase has emerged from the immunization studies where vaccination with recombinant *P. falciparum *enolase (r-Pfen) resulted in partial protection against *P. yoelii *induced malaria in mice and the detection of anti-enolase antibodies in human serum samples from malaria endemic region of India [32]. To examine the involvement of a housekeeping protein in diverse cellular functions, one can probe its sub-cellular localization and identify the interactor proteins, as involvement of a protein in multiple functions invariably requires its recruitment to different sub-cellular compartments and interactions with different proteins. These possibilities have been examined here using *in situ *(IFA and IEM) and biochemical fractionation (sub-cellular fractionation, pull down assays) methods for determining the localization of enolase in *P. falciparum *and identify the interacting proteins by co-localization and gel analysis.

## Methods

### Materials

Mouse monoclonal antibodies against Pfg-27 [33] and mouse anti-Pf HSP70 antibodies [34] used here have been characterized earlier. Anti-Pfs-48/45 monoclonal antibody was obtained from the MR4. Rabbit anti-*P. falciparum *aldolase antibody and a preparation of *P. yoelii *sporozoites were a kind gifts from Prof. Victor Nussenzweig, Department of Pathology, N.Y. University Medical Centre, New York, USA, and rabbit anti-*Toxoplasma gondii *actin antibody was provided by Prof. David Sibley, Washington university, St. Louis, MO, USA. RPMI media were from GIBCO-BRL, NY, USA. Protease inhibitor cocktail was procured from Roche Applied Science, Indianapolis, IN, USA. Saponin was from Sigma Chemical Co., St Louis, MO, USA. Alexa Fluor 488 conjugated anti-rabbit and anti-mouse IgG and DAPI were from Molecular Probes, NJ, USA and Vectashield-mounting medium was from Vector laboratories, CA, USA

### r-Pfen purification and anti enolase antibodies

The preparation of recombinant 6 × His-tagged *P. falciparum *enolase (r-Pfen) and production of rabbit and mouse anti-enolase antibodies were carried out as described earlier [35]. GST tagged r-Pfen was purified using glutathione-sephadex beads. For the *in situ *localization studies, the polyclonal anti-r-Pfen antibodies used have previously been shown to have high specificity against parasite enolase protein and did not cross react with homologous host enolases or other parasitic proteins under the experimental conditions employed here [32].

### Preparation of different stages of the parasite

*Plasmodium falciparum *asexual and gametocyte cultures (3D7 and NF54 isolates) were maintained as described previously [36,37]. The growth rates and proportions of various developmental stages of asexual and sexual parasites were monitored daily by microscopic examination of Giemsa-stained blood smears. Culture enriched with gametocytes was prepared and smears were made. Starting with synchronized asexual parasites grown in suspension culture as described, gametocytes were prepared by daily media changes of static cultures at 37°C.

### Indirect immunofluorescence assay (IFA)

Immunofluorescence assay was performed at room temperature with the air-dried blood smears. Cells were fixed with 4% formaldehyde in phosphate buffered saline (PBS) for 10 minutes, washed five times, permeabilized with 0.25% triton x-100 in PBS for 10 minutes, washed, fixed with 3% BSA-PBS for 45 minutes, incubated for 1 hour with the following antibodies combination: (i) mouse anti-enolase antibodies (1:200) with rabbit anti-*P. falciparum *aldolase (1:100) or (ii) rabbit anti-enolase antibodies (1:200) with mouse anti-HSP70 antibody (1:100). All antibody dilutions were made into 1% BSA-PBS. After antibody treatment, smears were washed 7–8 times with PBS. These smears were then treated with the secondary antibodies for 45 minutes (Alexa Fluor 488-conjugated anti-mouse IgG and Alexa Fluor 568-conjugated anti rabit IgG were used as secondary antibodies (1:500)) and washed 10–12 times with PBS. Parasite nuclei were stained with DAPI (1 μg·ml^-1^) and mounted with vectashield.

### Soluble and pellet fraction of asexual and gametocyte stages

Two separate preparations of erythrocytes, mostly infected with mature (stage IV-V) gametocytes and asexual stages of *P. falciparum *were harvested using saponin. For asexual stages, *P. falciparum *culture was allowed to reach ~5% parasitaemia, harvested and washed with incomplete RPMI solution. The gametocytes culture was similarly harvested and it consisted mostly of stage IV and V parasites with minor contamination (<3%) from mixed asexual stage parasites. Infected erythrocytes from both cultures were then treated with 0.05% saponin for 10 min at 4°C to release the parasites from the host erythrocyte membrane [38]. The parasite pellets were then solubilized in NETT buffer (10 mM Tris-HCl, pH 7.4, 150 mM NaCl, 1 mM EDTA, 0.5% Triton x-100) with cocktail protease inhibitor at 4°C for 10 minutes and centrifuged at 20,000 g for 30 minutes. The supernatant and pellets were used for SDS-PAGE and immunoblot analysis. Two dimensional gel electrophoresis and Western blotting for the visualization of the *P. falciparum *enolase variants was performed as described earlier [27].

### Cytochalasin-D treatment of parasites

*Plasmodium falciparum *culture containing wells were treated with cytochalasin-D dissolved in DMSO at a final concentration of 50 μM. Briefly, 5μl of 2 mM stock of cytochalasin D in DMSO was mixed with 95 μl of complete RPMI (100 μl of 100 μM cytochalasin-D), which was added to 100 μl of culture to obtain 50 μM final concentration of the drug. Only DMSO was added to the control wells. DMSO was kept at < 0.1% (v/v) under the experimental conditions. These were then incubated for 4 hours at 37°C. The cells were spun in the pre-warmed tubes and supernatant was removed to keep ~50% haematocrit. Thin smears were prepared, air dried and used for indirect immunofluorescence assay.

### Treatment with Triton X-100 prior to fixation

Detergent extraction was carried out prior to the fixation of the cells to remove the cytosolic and membrane associated proteins. *Plasmodium falciparum*-infected blood was smeared on poly-Lysine coated glass slides, which were then treated with 1% Triton x-100 for 10 minutes at room temperature (20°C). These were then washed three times with phosphate buffer saline and used for indirect immunofluorescence assay. This allowed cytoskeleton associated enolase to be visualized.

### Immuno-gold electron microscopy (IEM)

Preparations of *P. falciparum*-containing red blood cells were fixed in 4% paraformaldehyde (Electron Microscopy Sciences, PA) in 0.25 M HEPES (pH 7.4) for 1 hr at room temperature, then in 8% paraformaldehyde in the same buffer overnight at 4°C. They were infiltrated, frozen and sectioned as previously described [39]. The sections were immuno-labeled with mouse anti-r-Pfen antibodies (1:100 in PBS/1% fish skin gelatin), then with anti-mouse IgG antibodies, followed directly by 15 nm protein A-gold particles (Department of Cell Biology, Medical School, Utrecht University, the Netherlands) before examination with a Philips CM120 Electron Microscope (Eindhoven, the Netherlands) under 80 kV.

### Interaction of enolase with actin, tubulin and human plasminogen

For detection of direct interaction of enolase with actin (or tubulin), GST tagged-r-Pfen was immobilized on glutathione beads and was mixed with G-actin or tubulin. Samples were incubated for 2 hours at 37°C and subjected to centrifugation. Beads were washed with G-actin buffer (5 mM Tris-HCl pH 8.0, 0.2 mM CaCl_2_, 0.1 mM ATP) or tubulin buffer (80 mM Na-PIPES pH 6.9, 1 mM MgCl_2_, 1 mM EGTA, 1 mM GTP) respectively and analysed on a 12% SDS PAGE.

Binding of enolase with plasminogen was investigated using ELISA. Wells were coated with 100 μl of 100 nM plasminogen (or rabbit muscle pyruvate kinase). After over coating with skimmed milk and washing, 100 μl of 6 × His-tagged r-Pfen in different concentrations was added and incubated for 4 hours. Mouse anti r-Pfen serum (1:2000) was used in the assay. Remaining steps were same as described earlier [35].

## Results

### Expression levels of enolase at different stages of *P. falciparum*

**Figure 1:**
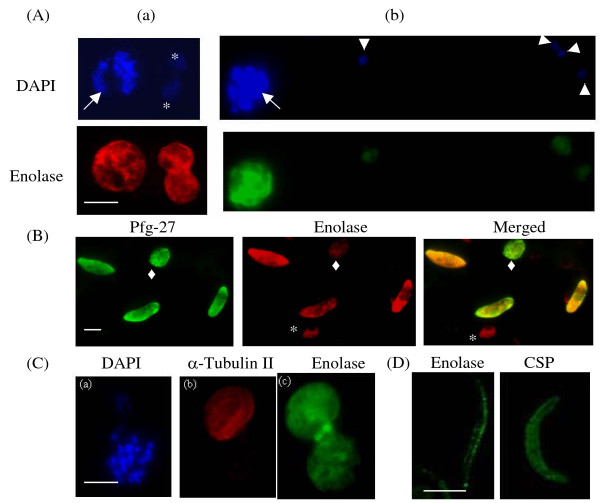
**Immunofluorescence assays (IFA) for the localization of enolase at different stages of *Plasmodium *life cycle**. (A) Asexual stages, (B) Gametocytes, (C) Gamete (mosquito stage) and (D) Sporozoite (mosquito salivary gland stage). Sporozoite preparation was from *P. yoelii*, while all other stages shown are for *P. falciparum*. Ring stages (triangle), trophozoite (*), schizont (→) and stage II gametocyte (diamond). Different fluorescent probes used were, (i) DAPI as a nuclear marker, (ii) mouse anti-r-Pfen (green), (iii) rabbit anti-r-Pfen (red), (iv) mouse anti-Pfg-27 (green) as gametocyte marker, (v) rabbit anti-male gametocyte specific α-tubulin II antibody (red) and (vi) mouse anti-CSP antibody (green) as sporozoite surface marker. The scale bars shown corresponds to 5 μm.

IFA analysis at different stages in the life cycle of *P. falciparum *is shown in Figure 1. Expression levels of enolase appear to be very similar in most stages except in certain sub-stages of the gametocyte. Figure 1A (a) shows schizont and trophozoite stages of the parasite. One of them is a multinucleated schizont stage (marked with →) and the other two are trophozoites (marked with *). Figure 1A (b) shows a schizont and four ring stage cells (marked with ▶). Figure 1B shows the distribution of enolase in the gametocyte stages. From the shape of the gametocytes, it appears that three elongated cells represent mature gametocytes (stage IV/V), whereas the small round, Pfg-27 positive cell may be an early stage II gametocyte (marked with ◆). Although Pfg-27, a gametocyte marker [40] appears to express equally in the various gametocyte sub-stages, the merged image showed that the stage II gametocyte has relatively lower levels of expressed enolase. In general, it was also observed that the levels of enolase are comparable at the gametes and the schizont stages (Figure 1C). The most unusual distribution was observed in sporozoite stage. Figure 1D shows a mosquito derived *P. yoelii *sporozoite preparation. Enolase staining in these cells showed a punctuate pattern where particulate enolase appears to be present beneath the plasma membrane, which is quite distinct from the homogenous staining observed for circum sporozoite protein (CSP).

### Sub-cellular distribution of enolase in *Plasmodium falciparum*

**Figure 2:**
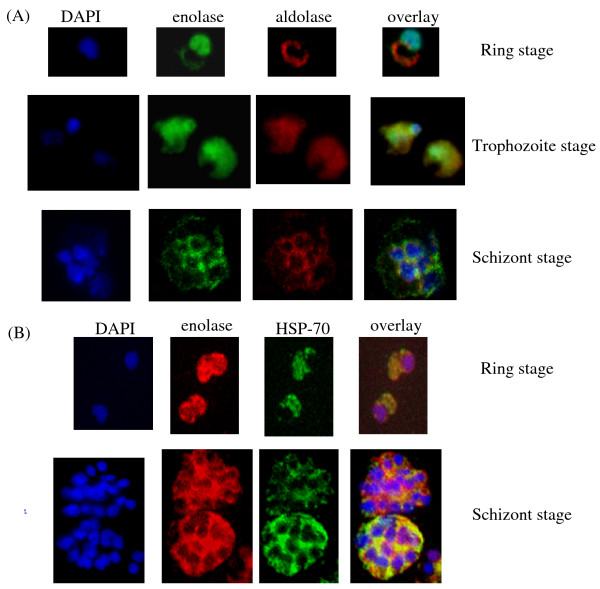
**Immunofluorescence assays for the localization of enolase, aldolase and HSP-70 in *P. falciparum *asexual stages (ring, trophozoite and schizont)**. (A) *P. falciparum *infected red blood cells were treated with DAPI (blue), mouse anti r-Pfen antibody (green), rabbit anti-*P. falciparum *aldolase antibody (red). (B) Cells were treated with DAPI, rabbit r-Pfen antibody (red), and mouse anti Pf HSP-70 antibody (green). Overlay panels show the merged of the three images.

**Figure 3:**
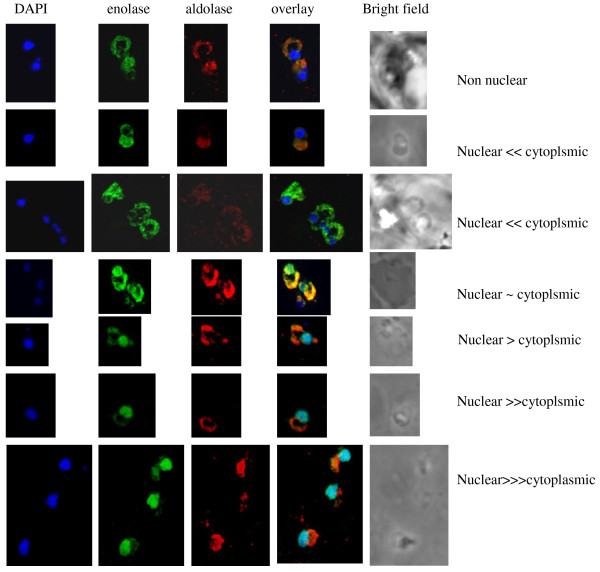
**Immunofluorescence images showing variation in distribution of enolase between cytosol and nucleus in a population of synchronized ring stage parasite cells**. There were greater number of cells having more enolase signal arising from nucleus than from cytosol.

In addition to cytosolic localization of enolase, its presence in the nucleus was observed in the ring and trophozoite stages (Figure 2). The possibility of spill over of cytosolic enolase into the nucleus during the processing of parasite cells could be ruled out by observing the localization of two other proteins, namely aldolase (Figure 2A) and HSP-70 (Figure 2B). Both these proteins were present in the cytoplasmic compartment and no nuclear presence was detected. In a synchronized population of ring stage parasites, wide variation in the relative distribution of enolase between the nuclear and the cytosolic compartments was observed. Certain cells had a faint staining for nuclear enolase, indicating that cytosolic enolase is much greater than the nuclear one while others had strong nuclear staining for the enolase (cytosolic << nuclear) (Figure 3).

**Figure 4:**
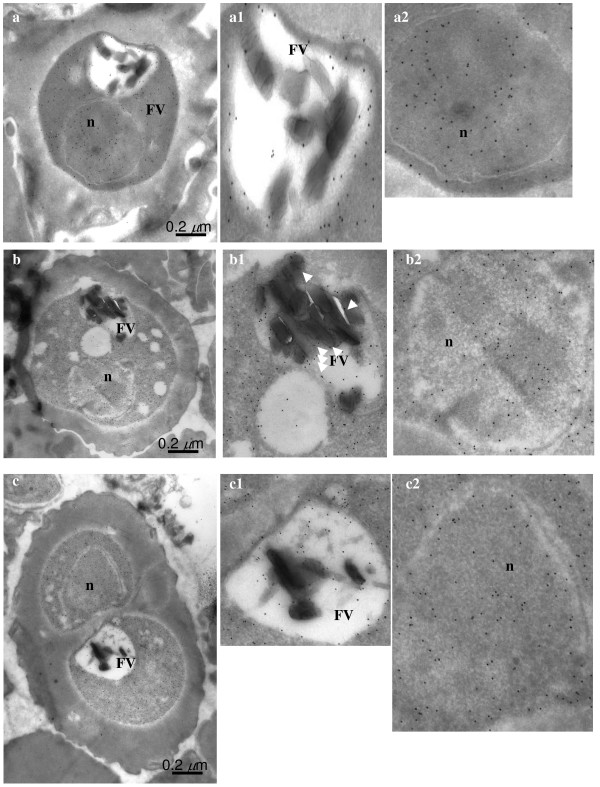
**Immuno-gold electron microscopic (IEM) imaging for the localization of enolase in early trophozoite satge of *P. falciparum *using mouse anti-r-Pfen antibody**. Magnified views of the food vacuole (FV) and nucleus (n) are also shown. Arrows in food vacuole marks hemozoin associated enolase.

**Figure 5:**
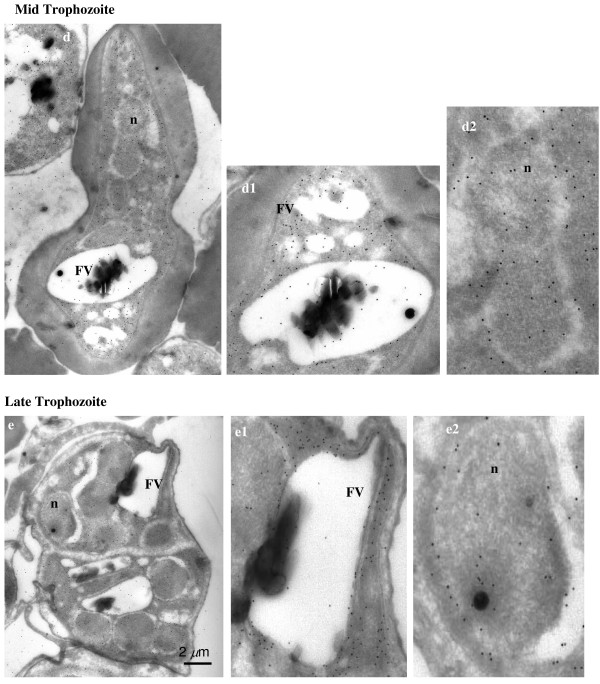
**Immuno-gold electron microscopic (IEM) imaging for the localization of enolase in mid and late stage trophozoites of *P. falciparum *using mouse anti-r-Pfen antibody**. Magnified views of the food vacuole (FV) and nucleus (n) are also shown.

**Figure 6:**
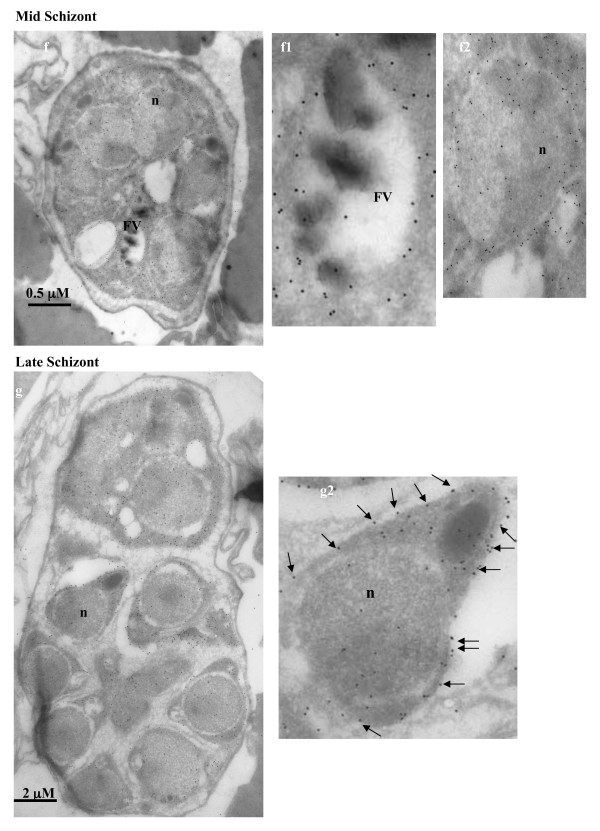
**Immuno-gold electron microscopic (IEM) imaging for the localization of enolase in mid and late stage schizonts of *P. falciparum *using mouse anti-r-Pfen antibody**. Magnified views of the food vacuole (FV) and nucleus (n) are also shown. Presence of enolase on the surface of a merozoite is marked with arrows.

**Figure 7:**
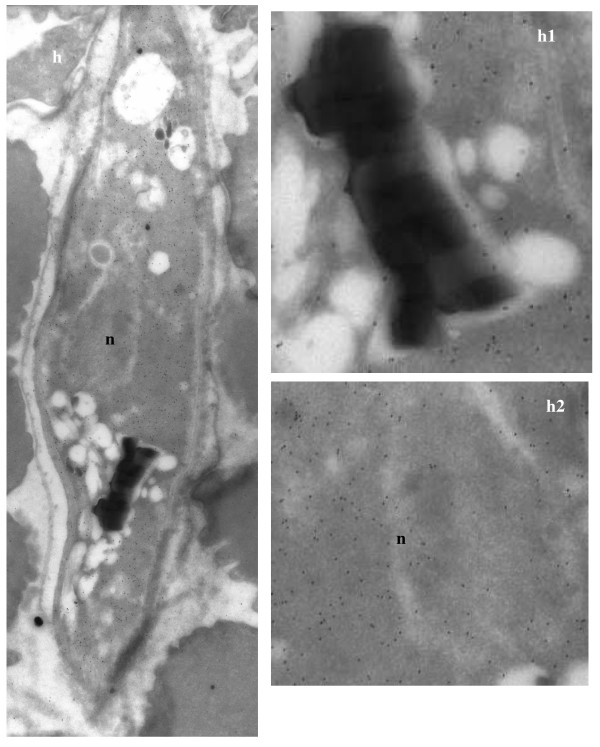
**Immuno-gold electron microscopic (IEM) imaging for the localization of enolase in gametocyte of *P. falciparum *using mouse anti-r-Pfen antibody**. Magnified views of the food vacuole (FV) and nucleus (n) are also shown.

In order to obtain better resolution, the sub-cellular localization of enolase in *P. falciparum *was also examined by immuno-gold electron microscopy (IEM). Figures 4, 5, 6, 7 show the parasite cells in the trophozoite, schizonts and gametocyte stages labeled with anti-r-Pfen. For all the images presented, a magnified view of food vacuole (FV) and nucleus (n) is shown along side. The nucleus and food vacuole have significantly higher levels of enolase present in early (Figure 4) and mid stage trophozoites (Figure 5) as compared to the late stage trophozoite (Figure 5) and the schizont (Figure 6). Nuclear enolase at gametocyte stage is also low as compared to cytosol (Figure 7). These observations are similar to the pattern of enolase distribution between nucleus and cytosol observed using IFAs (Figure 2). It is interesting to note that in all the stages observed in these IEM images, nuclear enolase exhibited a preferential association with electron-dense heterochromatin region (darker regions in the nucleus). Cell surface localization of enolase was observed at the merozoite stage, (Figure 6 g2). Further, in all these images there was no observable association of Pfen with infected host cell cytosol or cell membrane, suggesting that Pfen is not secreted into the cytosol or translocated to the cell membrane of the infected rbcs. This observation is at variance from the earlier reports [41].

### Association of enolase with the cytoskeletal elements of the parasite

**Figure 8:**
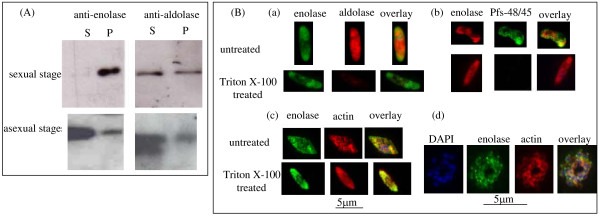
**(A) Comparison of the distribution of enolase, aldolase and actin in soluble and particulate fractions prepared from *P. falciparum *cells in sexual and asexual stages**. Cells were treated with 0.5% Triton X-100 for 10 minutes at 4°C. Solubilized proteins were removed by centrifugation (10,000 g for 30 minutes). Soluble (S) and particulate (P) fractions were analyzed on 12% SDS-PAGE and the presence of enolase was detected by Western blotting. (B) IFA of *P. falciparum *gametocyte stages (a, b, c) and asexual schizont stage (d). Cells were fixed either after a treatment with 1% Triton X-100 for 10 minutes (for the removal of cytosolic and membrane proteins) or without Triton treatment. Fixed cells were stained with rabbit or mouse anti-r-Pfen antisera along with (a) rabbit anti-*P. falciparum *aldolase (red), (b) mouse anti Pfs48/45 (green), (c) rabbit anti-*T. gondii *actin antibody (red) and (d) schizont (asexual stage) stained with DAPI, anti-r-Pfen and anti-actin antibodies after detergent treatment.

Parasite cell extracts prepared with Triton-X-100 (TX100) as the solubilizing agent, were analyzed on SDS-PAGE and enolase was visualized by western blotting. Figure 8(A) presents the distribution of the enolase between the soluble and particulate fractions in the sexual and asexual stages of the parasite life cycle. While in asexual stages, >80–90% enolase was present in TX100 soluble fraction and only ~10–20% was associated with cytoskeletal components, in sexual stages the distribution was quite the opposite. Most of the enolase in sexual stages was associated with detergent insoluble fraction. In contrast, aldolase was equally distributed between soluble and pellet fractions in sexual stages, while its distribution in asexual stages was more like enolase (soluble >> pellet). Observation of differential distributions of aldolase and enolase in soluble and particulate fractions, ruled out the possibility that such results may arise from incomplete lysis of cells or cross contamination of particulate fraction with the soluble proteins.

**Figure 9:**
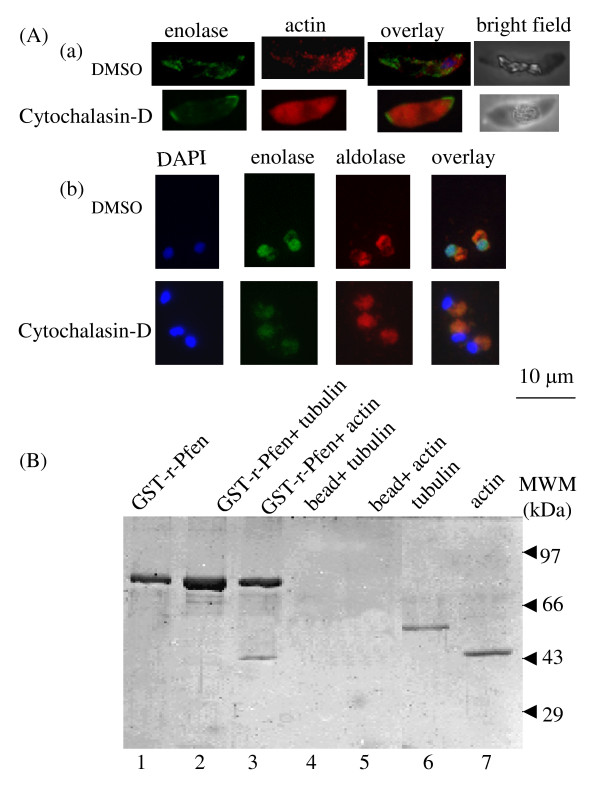
**Demonstration of actin association with enolase in *P. falciparum*: (A) Effect of cytochalasin D (actin depolymerizing drug) on sub-cellular distribution of enolase in (a) gametocyte (sexual stage) and (b) rings (asexual stage)**. Cells were treated with 50 μM cytochalasin D or DMSO (control) and IFA was performed with anti-r-Pfen antibodies (green) and rabbit anti-*T. gondii *actin antibody (red). Disruption of actin cytoskeleton led to accumulation of enolase at the two ends of the gametocyte cell (a), whereas in asexual stage translocation of enolase to nucleus was disrupted (b). (B) Direct interaction of GST tagged-r-Pfen with rabbit muscle G-actin and tubulin. GST-r-Pfen was adsorbed on glutathione-sepharose beads and was incubated with G-actin or tubulin for 2 hours at room temperature. Beads were collected by centrifugation and washed with appropriate buffers. The samples were analyzed on 12% SDS-PAGE. Third lane (from left) showed a pull down of G-actin with r-Pfen.

For the *in situ *observation of the distribution of enolase and aldolase in the gametocytes, immunofluorescence assays were performed on TX100 treated and untreated cells (Figure 8B(a)). Although both enolase and aldolase are glycolytic enzymes, they show very different distribution in gametocyte stages as seen from fractionation as well as IFA studies. Since cytosol has considerable amount of enolase, it was important to ensure that the detergent treatment did remove cytosolic and the membrane associated enolase. This was evident from the disappearance of signals for aldolase (a cytosolic protein) (Figure 8B(a)) and a gametocyte membrane protein Pfs48/45 (Figure 8B(b)) in the detergent treated preparations. Figure 8B shows a gametocyte (Figure 8B(c)) and a schizont (Figure 8B(d)) stained with DAPI, anti-enolase and anti-actin antibodies, before and after the treatment with TX100. Co-localization of enolase with actin is observed in the gametocyte stage while it is rather sparse in the schizont stage. Disruption of actin cytoskeleton by treatment with cytochalasin D resulted in disruption of actin co-localization pattern of enolase in gametocytes (Figure 9A(a)). Such a treatment of ring stage parasites resulted in the loss of translocation of Pfen to the nucleus (Figure 9A(b)).

Observations of co-localization of actin and enolase as well as the effect of cytochalasin-D on enolase distribution suggested that two proteins may have direct interaction. Possibility of such an interaction was investigated by incubating GST tagged-r-Pfen (immobilized on glutathione beads) with G-actin or tubulin. Results are shown in Figure 9B. Pull-down assay did not show any interaction with tubulin, however direct binding of actin with enolase was observed (Figure 9B, lanes 3 & 5). In the spun-down preparations of parasite actin filaments, enolase along with several other proteins has been detected [42]. However, it was not evident from these studies, whether Pfen and actin have direct interaction.

### Enolase binds to human plasminogen

**Figure 10:**
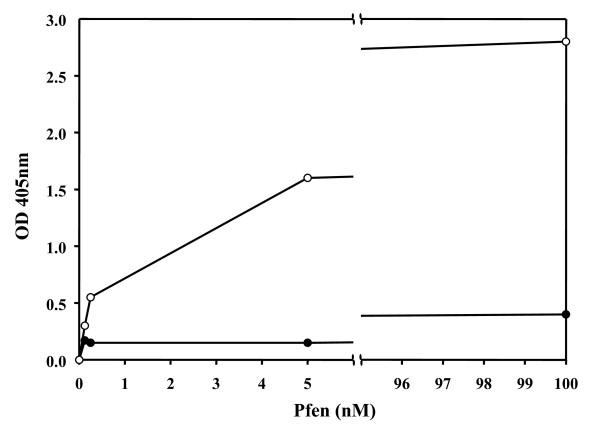
**Binding of *P. falciparum *enolase to human plasminogen**. ELISA plates were coated with 100 μl of 100 nM plasminogen (○-○) or rabbit muscle pyruvate kinase (●-●). Assay was performed as described in materials and methods. Binding of the r-Pfen to plasminogen is evident from the observed high OD at 405 nm as compared to pyruvatekinase (control).

In addition to cytosolic and nuclear presence, *Plasmodium spp *enolase has been shown to reside on the merozoite surface (Figure 6) [32]. In certain pathogenic bacteria, cell surface enolase has been shown to serve as plasminogen receptor. Through this interaction, these pathogens exploit the fibrinolytic activity of plasmin(ogen) to their advantage in tissue invasion [43]. In order to address whether Pfen also interacts with plasminogen, an ELISA assay was performed. A concentration dependent binding of Pfen with human plasminogen was observed in this assay. However, Pfen did not show any significant binding to rabbit muscle pyruvatekinase, which was used as a control (Figure 10).

## Discussion

Large scale stage specific analysis of *P. falciparum *proteome has shown that the enolase is expressed in all stages (Trophozoite, schizont, merozoite, gametocyte) in the life cycle of the parasite [44,45] and was also associated with the host cell plasma membrane of the infected red blood cells (iRBCs) [41]. The results presented here showed that indeed the Pfen is present in all the asexual and sexual stages of the parasite. However, in the IFA and IEM images presented here, there was no detectable Pfen associated with iRBC membranes or iRBC cytoplasm suggesting the possibility of cross contamination during biochemical sample preparations in the earlier studies. The validity of the results reported here heavily rely on the specificity of anti-Pfen antibodies to react only with *Plasmodium *enolase. In an earlier report, several controls were performed to ensure that indeed this was the case. It was shown that under the experimental conditions employed here, these antibodies did not show any cross reactivity towards protein extracts from uninfected RBCs, human & mouse leukocytes, and mouse liver in western analysis. Whole cell extracts from *P. yoelii *and *P. falciparum *showed a single band at an expected molecular mass of about 50 kDa [33]. The results presented here also indicate that the expressed levels of enolase protein do not match with the levels of transcript at various stages of the parasite reported earlier [46,47]. For instance, in sexual stages, gametocytes have negligible amounts of Pfen transcript and in sporozoites (mosquito salivary gland stage), Pfen transcript is undetectable [46-48]. However, the images presented here showed good quantities of Pfen protein in both gametocytes and sporozoites (Figure 1). These results suggest the possibility of expressed protein levels of enolase in the parasite being controlled by post-transcriptional regulation of translation.

The observed presence of Pfen in multiple sub-compartments of the parasite cell may imply multiple physiological functions for this protein. In the asexual stages, it is largely soluble while in the sexual stages, it is mostly particulate (Figure 8A). It is possible that most of the enolase in asexual stages is recruited for glycolytic function, while in gametocyte stages, it may have non-glycolytic functions. Punctate appearance of Pfen close to the sporozoite surface (Figure 1D) also indicates additional non-glycolytic function for this protein. *Plasmodium *invades tissues at sporozoite (liver cells), merozoite (RBCs) and ookinete (mosquito gut wall) stages. Results presented here showed that the Pfen binds to human plasminogen and is surface localized in merozoite stage. Two signatures of α-enolase have been proposed for such interaction with plasminogen, namely the Lysine residues at the C-terminal end and in the central motif ^257^DLDFKSDDPS [24,49]. The C-terminal Lysine residue and the central motif (^266^DLDFKTPNNDKS in Pfen) are both conserved in *P. falciparum *enolase [35], and therefore the binding to plasminogen is expected. The presence of plasminogen receptors on cell surface and the non-fibrinolytic functions of plasminogen have now been documented extensively [50-52]. The nonfibrinolytic roles of plasminogen seem to depend on its ability to activate matrix metalloproteinases, which degrade matrix proteins [53]. Such plasmin-dependent pericellular proteolysis may operate when plasmin(ogen) is tethered to the cell surface through a heterogeneous group of plasminogen receptors, and enolase is documented to be one such receptor [24,49]. It is possible that surface localized enolase in *Plasmodium *zoite forms may also act as a plasminogen receptor and play a role in tissue invasion processes. This is consistent with the observation that the anti-Pfen antibodies block merozoite invasion into red cells [32].

Glycolytic proteins have been reported to perform diverse functions at different sub-cellular locations. *Plasmodium *aldolase has glycolytic function in cytosol while in association with acto-myosin complex, it assists the parasite in invasion and motility functions [8]. In a proteome-wide yeast two hybrid screen La count *et al *[54], identified glyceraldehydes-3-phosphate dehydrogenase (PF14_0598), MSP-9 (PFL1385c), cysteine proteinase (PFB0330c) and formin (PFL0925w) as direct interactors of enolase. These authors also reported HSP-70 (PF11_0351) to be an indirect interactor. Observation of direct binding of Pfen and actin as also the extensive association of enolase with the particulate fraction (especially in the sexual stages) implies a role for enolase in the cytoskeletal organization. In a recent study where protein modifications occurring under oxidative stress were assessed, Pfen was found to undergo major modifications [55] indicating that it is likely to be a target protein for stress response.

In *P. falciparum*, presence of relatively high amounts of nuclear Pfen was observed in the ring and the early trophozoite stages (Figures 2, 3 &4). In IEM images, preferential association of enolase with heterochromatin was noted, particularly in early trophozoite (or ring) stages (Figure 4a2 &4b2). Translocation of cytosolic enolase to nucleus, just when the growth phase begins is suggestive of possible involvement of enolase in transcription related processes. Possibility of such nuclear function for enolase has been suggested in mammalian [56] and plant cells [57]. The nuclear presence of enolase has also been reported in closely related apicomplexan *Toxoplasma gondii *and *Eimeria tenella*. However, a direct correlation between translocation of Pfen and transcriptional regulation has not yet been demonstrated in parasites [28,29].

Food vacuole in *Plasmodium *is an acidic proteolytic compartment central to the metabolism of the parasite [58]. In Figure 4, magnified IEM images of vacuolar region of *P. falciparum *showed close association of enolase with haemozoin (marked with arrow heads in Figure 4b1). In the growing trophozoite (Figures 4 and 5), there was considerable amount of enolase present in the vacuole. However, in the mature trophozoite (Figure 5) and schizont stages (Figure 6), the amounts of enolase associated with FV seemed to decrease (although cytosolic enolase is quite abundant), suggesting a role in early stages of vacuolar development and/or haemozoin formation. The observed pattern of enolase association with vacuole appeared very similar to the proteins which have been implicated in haem detoxification and vacuole biogenesis [59,60] raising the possibility of enolase being involved in such functions. Further support for possible involvement of enolase in haem detoxification arises from the observed association with ferriprotoporphyrin IX (FPIX), prepared from chloroquine treated parasites [61]. Other possible vacuolar function for Pfen can be its involvement in vacuolar fusion and vacuolar protein sorting as observed in yeast [25].

In eukaryotic cells multi-compartment localization of a protein synthesized in cytoplasm is achieved by compartment specific topogenic sequences. *Plasmodium falciparum *enolase does not have any such signal sequences to be targeted to nucleus or cell surface membrane. In *Plasmodium*, there are no signal sequence(s) known, which ensure targeting to food vacuole either. Post-translational modifications and association with other interactors provide alternative mechanisms for such diverse localization [62]. Additional studies are needed to identify protein interactors of enolase, post-transcriptional modifications that it undergoes and whether it has any function in nucleus and food vacuole of the parasite.

## Conclusion

The results presented in this paper provide evidence for multiple subcellular localization of enolase and the stage specific variation in the levels of the expressed enolase protein. *P. falciparum *enolase exhibits great diversity in sub-cellular localization with stage specific variation viz-a-viz – a) Presence of enolase on merozoite surface; b) ability of anti-r-Pfen antibodies to interfere with invasion process and accord partial protection against malaria; c) nuclear and vacuolar localization and observed shift between soluble to particulate fractions in asexual and sexual stages. These variations are indicative of the involvement of *P. falciparum *enolase in a host of biological functions. Association of enolase with actin appears to be important for its translocation to nucleus. An analysis of the nature of interactions of enolase with its interactor proteins and post-translational modifications that it undergoes, may provide insights in to the molecular basis for the multiple physiological functions that this protein might perform in the parasite.

## Abbreviations

DAPI: 4',6-Diamidino-2-phenylindole; IFA: immuno fluorescence assay; IEM: immuno-gold electron microscopy; r-Pfen: recombinant *P. falciparum *enolase (EC 4.2.1.11); TX100: Triton-X-100.

## Competing interests

The authors declare that they have no competing interests.

## Authors' contributions

IPB, SS, GKJ had the idea and designed the study. IPB, IC, NK, GKJ performed all the experiments. IPB, SS, GKJ wrote the manuscript. All authors participated in interpretation of results, read and approved the final manuscript.
